# Activation of a [NiFe]-hydrogenase-4 isoenzyme by maturation proteases

**DOI:** 10.1099/mic.0.000963

**Published:** 2020-07-30

**Authors:** Alexander J. Finney, Grant Buchanan, Tracy Palmer, Sarah J. Coulthurst, Frank Sargent

**Affiliations:** ^1^​ School of Natural & Environmental Sciences, Faculty of Science, Agriculture & Engineering, Newcastle University, Newcastle upon Tyne NE1 7RU, UK; ^2^​ School of Life Sciences, University of Dundee, Dundee DD1 5EH, Scotland; ^3^​ Institute of Biosciences, Faculty of Medical Sciences, Newcastle University, Newcastle upon Tyne NE1 7RU, UK

**Keywords:** *Escherichia coli*, *Pectobacterium atrosepticum*, formate hydrogenlyase, hydrogenase, maturase, protease

## Abstract

Maturation of [NiFe]-hydrogenases often involves specific proteases responsible for cleavage of the catalytic subunits. *Escherichia coli* HycI is the protease dedicated to maturation of the Hydrogenase-3 isoenzyme, a component of formate hydrogenlyase-1. In this work, it is demonstrated that a *Pectobacterium atrosepticum* HycI homologue, HyfK, is required for hydrogenase-4 activity, a component of formate hydrogenlyase-2, in that bacterium. The *P. atrosepticum* Δ*hyfK* mutant phenotype could be rescued by either *P. atrosepticum hyfK* or *E. coli hycI* on a plasmid. Conversely, an *E. coli* Δ*hycI* mutant was complemented by either *E. coli hycI* or *P. atrosepticum hyfK in trans. E. coli* is a rare example of a bacterium containing both hydrogenase-3 and hydrogenase-4, however the operon encoding hydrogenase-4 has no maturation protease gene. This work suggests HycI should be sufficient for maturation of both *E. coli* formate hydrogenlyases, however no formate hydrogenlyase-2 activity was detected in any *E. coli* strains tested here.

## Full-Text

Hydrogenases are enzymes that are widespread in microbial systems where they catalyse the oxidation or production of molecular hydrogen (H_2_) [1]. A major class of hydrogenases common in Proteobacteria are the [NiFe]-hydrogenases that rely on an elaborate Ni-Fe-CO-2CN^-^ metallocofactor at their active sites [1]. These two-part enzymes, consisting of a large subunit (~60 kDa) harbouring the [NiFe]-cofactor and a small subunit (~30 kDa) that contains iron-sulfur clusters, require the coordination of both specific and housekeeping biosynthetic pathways for their assembly and activation [2]. The biosynthesis pathway of the large subunit includes the critical final steps of cofactor assembly and insertion. Here, the HypA and HypB accessory proteins insert the nickel ion in to the large subunit as the final component of the [NiFe]-cofactor, where the HypA monomer interacts with the unstructured N-terminus and a C-terminal beta strand of the immature large subunit [3]. This novel interaction brings the HypA nickel binding site and immature hydrogenase large subunit active site in proximity to allow nickel transfer [3]. Next, and for the vast majority [NiFe]-hydrogenases, one final maturation step is required before small subunit docking and full enzymatic activation can occur. This is the proteolytic cleavage of a short stretch of polypeptide from the C-terminus of the hydrogenase large subunit [4].

Proteolytic maturation of hydrogenases is well understood for the model *Escherichia coli* [NiFe]-hydrogenase-3 isoenzyme (Hyd-3). Here, the Hyd-3 large subunit (encoded by the *hycE* gene) is cleaved after residue Arg-537 by a specific metallopeptidase termed HycI [5–8]. Removal of the 32-residue C-terminal ‘assembly peptide’ from HycE results in essentially irreversible cofactor-loading, correct folding of the large subunit and successful docking of the small subunit to generate the final active Hyd-3 [2]. Deletion of the *hycI* gene in *E. coli* led to the complete loss of all Hyd-3 activity and accumulation of an immature, unprocessed version of HycE [9]. Subsequent research in numerous other biological systems resulted in the central dogma that, where [NiFe]-hydrogenase large subunits were synthesized with a C-terminal extension or assembly peptide, that they would require processing by a specific protease for activation, and that said protease would be encoded close to the gene for the large subunit, and that said protease would not normally recognize any other hydrogenase homologues [4].

**Fig. 1.**
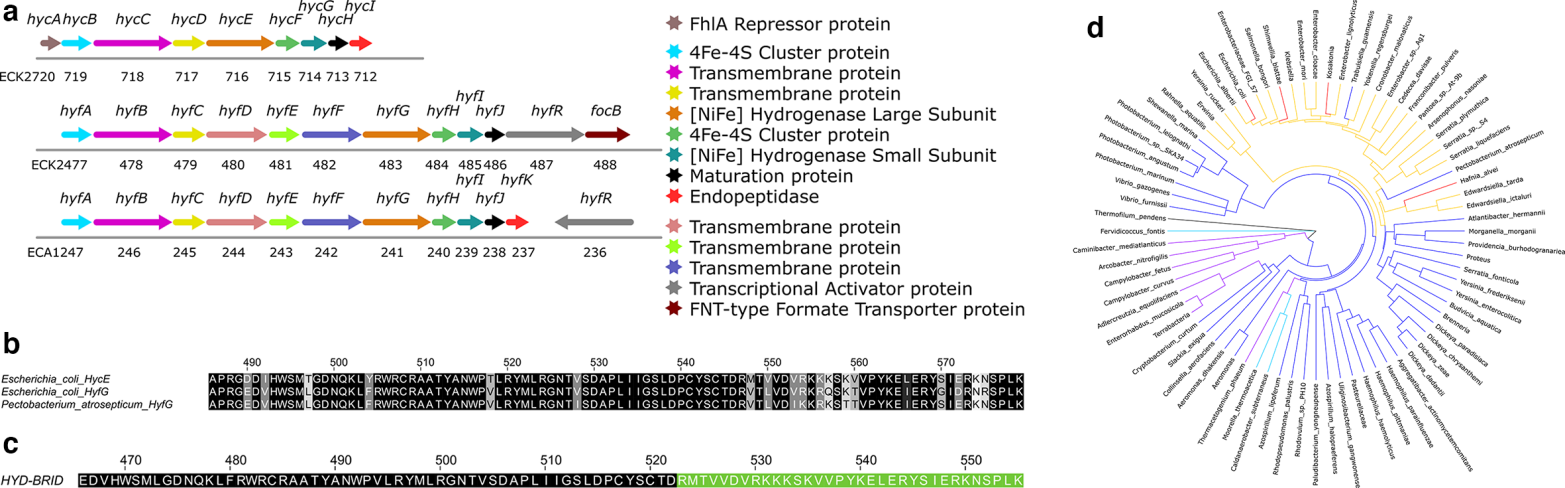
The genetics of processing group 4A [NiFe]-hydrogenases (a) Schematic showing the genetic organization of *E. coli* K-12 *hyc* and *hyf* operons as well as *P. atrosepticum* SCRI1043 *hyf* operon. Gene products are indicated in the legend and colour coded. (Top) The *E. coli* K-12 *hycABCDEFGHI* operon comprises genes ECK2720-ECK2712. (Middle) The *E. coli* K-12 *hyfABCDEFGHIJRfocB* operon comprises genes ECK2477-ECK2488. (Bottom) The *P. atrospecticum* (formerly *Erwinia carotovora *) *hyfABCDEFGHIJK-hyfR* cluster comprises genes ECA1247-ECA1236. The *hycI*- or *hyfK*-like genes are highlighted in red. (b) A sequence alignment of the final 93 amino acids, including the proteolytically processed assembly peptides, of HycE*_Ec_*, HyfG*_Ec_* and HyfG*_Pa_* with black to white shading showing most to least conserved residue positions. Sequence alignment was performed using Clustal [45] and presented using BOXSHADE (http://sourceforge.net/projects/boxshade/). (c) The hydrogenase hybrid ('HYD-BRID') C-terminal sequence of the ϕHyfG-HycE*_Ec_* construct introduced into *E. coli* FTE001, FTE002, FTE004 and FTE007 strains (Table 1). The sequence coloured black corresponds to the relevant part of the *E. coli* HyfG protein, and the sequence shaded green is the terminal arginine of the mature enzyme and the C-terminal assembly peptide of *E. coli* HycE. (d) A phylogenetic tree of all group 4A [NiFe]-hydrogenase-associated endopeptidases. Homologues were identified using blast [46] before multiple sequence alignment was carried out in Jalview [47]. Phylogenetic trees were constructed using FigTree (http://tree.bio.ed.ac.uk/software/figtree/). Dark blue and yellow colouring highlight those organisms with maturation protease genes associated with *hyf* and *hyc* type group 4A subtypes, respectively. Purple and cyan colouring shows organisms with an additional removal or variation in position of the *hyfD* gene, respectively (occurs within the *hyf* type only). Red colouring highlights organisms with both *hyc* and *hyf* operons (but note that these harbour only one maturation protease gene within their *hyc* operons). Note that this sequence analysis identified a HyfK homologue in *Trabulsiella guaensis*, which produces a functional Hyd-4 [29].

*E. coli* Hyd-3 is a member of the group 4A [NiFe]-hydrogenases [10] and a component of the formate hydrogenlyase-1 (FHL-1) complex [11]. It is encoded within the *hycABCDEFGHI* operon that includes the gene for the protease [6, 12]. Interestingly, laboratory strains of *E. coli* encode two separate group 4A [NiFe]-hydrogenases, each predicted to be part of distinct formate hydrogenlyase complexes. Thus in addition to Hyd-3, the *E. coli* [NiFe]-hydrogenase-4 isoenzyme (Hyd-4) is encoded by the *hyfABCDEFGHIJR-focB* operon [13] and is predicted to be a component of a formate hydrogenlyase-2 (FHL-2) complex [11]. FHL-1 and FHL-2 share the same core architecture, with FHL-2 predicted to contain extra membrane-embedded components [11]. FHL activity is normally maximal under fermentative conditions when the enzyme catalyses the oxidation of formic acid and couples this directly to the reduction of protons to molecular H_2_. Thus group 4A [NiFe]-hydrogenases have a physiological role in the evolution of hydrogen gas [11]. Directly demonstrating the enzymatic activity of *E. coli* FHL-2 or Hyd-4 has proven challenging. Under laboratory conditions, the enzyme appeared to be neither transcribed nor enzymatically active [14–16], although there is some evidence for a physiological role in H_2_ metabolism under some specific environmental conditions [17, 18]. In addition, disruption of Hyd-4 genes alone did not affect overall H_2_ production by *E. coli* [19, 20], again indicating that cellular Hyd-4 activity was very low or absent under the conditions tested. It is also clear that the *E. coli hyfABCDEFGHIJR-focB* operon does not encode any homologue of HycI (Fig. 1a) nor any other protease [2, 13]. Therefore, while the large subunit of Hyd-4 (HyfG) shares a high degree of sequence identity with HycE including the presence of a 32-residue C-terminal assembly peptide (Fig. 1b), it must also be considered that the apparent low activity of Hyd-4 may stem from incomplete maturation of the enzyme.

In this work, we set out to test the initial hypotheses that the *E. coli hyf* operon is not sufficiently expressed, and that HyfG is not correctly processed, such that a hydrogenase-null phenotype is observed. To do this we took a recombineering approach and constructed 15 new strains (Table 1 and Supplementary Material, available in the online version of this article) with alternative promoters and/or ϕ*hyfG::hycE* fusion alleles at the native *hyf* locus on the chromosome. None of the new strains displayed any Hyd-4 activity (Table 1). Briefly, a group of *E. coli* strains with modified *hyf* transcriptional promoter regions were generated using P1 phage transduction [21] and allelic exchange [22]. All engineering was carried out in single copy on the chromosome, and the strains’ ability to produce H_2_ gas under fermentative conditions was assayed by gas chromatography [23]. Initially, an *E. coli* K-12 strain (MG056G1, Table 1) was constructed based on the MG1655 parent strain [24] but encoding an internal 10-His tag between residues Gly-85 and Ala-86 within the HyfG protein. The rationale here was that a similarly modified version of HycE (Hyd-3) had retained full activity [25] and that the tag would allow further characterization of Hyd-4 at the protein level if the promoter engineering were successful. Next, the MG056G1 strain was further modified to replace the native *hyf* promoter region with that from the *E. coli hyc* operon encoding Hyd-3. This new strain (AF01, Table 1) was then extensively modified, first with the genetic removal of hydrogenase-3 activity (resulting in strains AF02 and AF03, Table 1), then by the additional deletion of the gene encoding the hydrogenase-1 catalytic subunit (yielding strains AF04 and AF06, Table 1). Culturing of all of these strains in triplicate 5 ml Lysogeny Broth (LB) supplemented with 0.8 % (w/v) glucose in sealed Hungate tubes for 16 h at 37 °C demonstrated that replacement of the *hyf* promoter region with that of *hyc* did not result in detectible H_2_ production from Hyd-4 (Table 1).

**Table 1.** Rational engineering of the *E. coli hyf* operon does not induce H_2_ production

* E. coli* K-12 strain	Relevant genotype	Source	H_2_ production
**MG1655**	F^-^, λ^-^, *rph-1*	[24]	Positive
**MG056G1**	as MG1655, *hyfG* ^His^	This Work	Positive
**AF01**	as MG1655, *hyfG* ^His^, P*_hyc_*::*hyfA*	This Work	Positive
**AF02**	as MG1655, *hyfG* ^His^, P*_hyc_*::*hyfA,* Δ*hycA-I*:: Kan^R^	This Work	Negative
**AF03**	as MG1655, *hyfG* ^His^, P*_hyc_*::*hyfA,* Δ*hycA-I*	This Work	Negative
**AF04**	as MG1655, *hyfG* ^His^, P*_hyc_*::*hyfA,* Δ*hycA-I,* Δ*hyaB*::Kan^R^	This Work	Negative
**AF06**	as MG1655, *hyfG* ^His^, P*_hyc_*::*hyfA,* Δ*hycA-I,*Δ*hyaB*	This Work	Negative
**FTE001**	as MG1655, *hyfG* ^His^, P*_hyc_*::*hyfA,* Δ*hycA-I*, ϕ*hyfG* (nt 1–1569)::*hycE* (nt 1611–1707)	This Work	Negative
**MG059e1**	as MG1655, *hycE* ^His^	[25]	Positive
**MGE1dI**	as MG1655, *hycE* ^His^, Δ*hycI*	This Work	Negative
**MC4100**	F^−^, *araD*139, Δ(*argF-lac*)169, λ^−^, e14^−^, *flhD*5301, Δ(*fruK-yeiR*)725(*fruA*25), *relA*1, *rpsL*150(Str^R^), *rbsR*22, Δ(*fimB-fimE*)632(::IS*1*), *deoC1*	[27]	Positive
**FTD147**	as MC4100, Δ*hyaB*, Δ*hybC*, Δ*hycE*	[16]	Negative
**AF05**	as MC4100, Δ*hyaB*, Δ*hybC*, Δ*hycE*, P*_hyc_*::*hyfA*	This Work	Negative
**FTE002**	as MC4100, Δ*hyaB*, Δ*hybC*, Δ*hycE*, P*_hyc_*::*hyfA*, ϕ*hyfG* (nt 1–1569)::*hycE* (nt 1611–1707)	This Work	Negative
**FTE003**	as MC4100, Δ*hyaB*, Δ*hybC*, Δ*hycE*, P_T5_::*hyfA*	This Work	Negative
**FTE004**	as MC4100, Δ*hyaB*, Δ*hybC*, Δ*hycE*, P_T5_::*hyfA*, ϕ*hyfG* (nt 1–1569)::*hycE* (nt 1611–1707)	This Work	Negative
**FTE005**	as MC4100, Δ*hyaB*, Δ*hybC*, Δ*hycE*, P_T5_::*hyfA*, *hyfG* ^His^	This Work	Negative
**FTE006**	as MC4100, Δ*hyaB*, Δ*hybC*, Δ*hycE*, P_T5_::*hyfA*, *hyfG* ^His^, ϕ*hyfG* (nt 1–1569)::*hycE* (nt 1611–1707)	This Work	Negative
**FTE007**	as MC4100, Δ*hyaB*, Δ*hybC*, Δ*hycE*, ϕ*hyfG* (nt 1–1569)::*hycE* (nt 1611–1707)	This Work	Negative

*E. coli strains were grown under anaerobic fermentative conditions in LB medium supplemented with 0.8 % (w/v) d-glucose at 37 °C for 16 h. Production of molecular H_2_ in the culture headspace was determined by gas chromatography. Hungate tube headspace gas was injected into a 500 µl loop and separated through a 5A molecular packed column before thermal conductivity detection. A hydrogen standard curve was generated using N_2_:H_2_ mixes [23].

LB, Lysogeny Broth.

Next, an alternative *E. coli* K-12 parental strain (based on MC4100 [26, 27]) was tested. The *E. coli* FTD147 strain (deleted for the genes encoding the catalytic subunits of Hyd-1, -2 and -3 [16]) was modified by replacement of the native *hyf* promoter with that of the strong T5 promoter from the pQE plasmid series (yielding strain FTE003, Table 1). Growth of this strain under fermentative conditions did not result in any detectible H_2_ production from Hyd-4 (Table 1).

Finally, it was considered that potential problems with HyfG processing could be leading to synthesis of an immature, inactive Hyd-4. This hypothesis is based on the fact that the *hyf* operon encodes no specific maturation protease and the reasonable possibility that HycI might not recognize HyfG as a substrate. In order to test this hypothesis, with the aim of forcing HycI to recognize and activate HyfG, a series of strains were carefully constructed where the C-terminal assembly peptide of HycE was added to the mature sequence of HyfG (Table 1, Fig. 1c). Careful genetic engineering generated a ϕ*hyfG::hycE* fusion sequence that would comprise the first 1569 nucleotides of *hyfG* precisely in-frame with *hycE* nucleotides 1611–1707 and retaining the ribosome binding site and initiation codon on the downstream *hyfH* gene to mitigate against potential polar effects. The resulting protein sequence is shown in Fig. 1c. This construct was transferred to the chromosome of a number of promoter-engineered strains (note that these all remain *hycI* ^+^) using the technique of homologous recombination [22]. No H_2_ production from Hyd-4 was detected in any of the engineered large subunit fusion strains (Table 1). Taken altogether, the strain-engineering experiments suggest that additional, unknown, biosynthetic problems are hindering assembly of *E. coli* Hyd-4.

Clearly, making progress in the understanding the biochemistry of Hyd-4-like enzymes requires an alternative model system. Recently, group 4A [NiFe]-hydrogenases from *Pectobacterium atrosepticum* [28], *Trabulsiella guaensis* [29], *Sulfurospirillum multivorans* [30], *Campylobacter concisus* [31] and *Parageobacillus thermoglucosidasius* [32] have been identified as possible candidates for study of this [NiFe]-hydrogenase group. Our sequence analysis suggests that genetic loci encoding each of these ‘*hyf*-type’ enzymes contained a *hycI*-like gene (Fig. 1d). Indeed, bioinformatic analysis of group 4A hydrogenases revealed only four organisms that encode both a Hyd-3 and Hyd-4 orthologue within their respective genomes: *Escherichia coli; Shimwellia blattae; Hafnia alvei*; and *Koskonia radicincitans* [11, 33]. In every case only one endopeptidase gene is found within the *hyc*-like operons, and none could be identified within the *hyf*-like operons (Fig. 1d, organisms linked by the red line). This suggests that a single HycI-like protease may be sufficient for Hyd-4 biosynthesis, given that a second copy is never conserved.

**Fig. 2.**
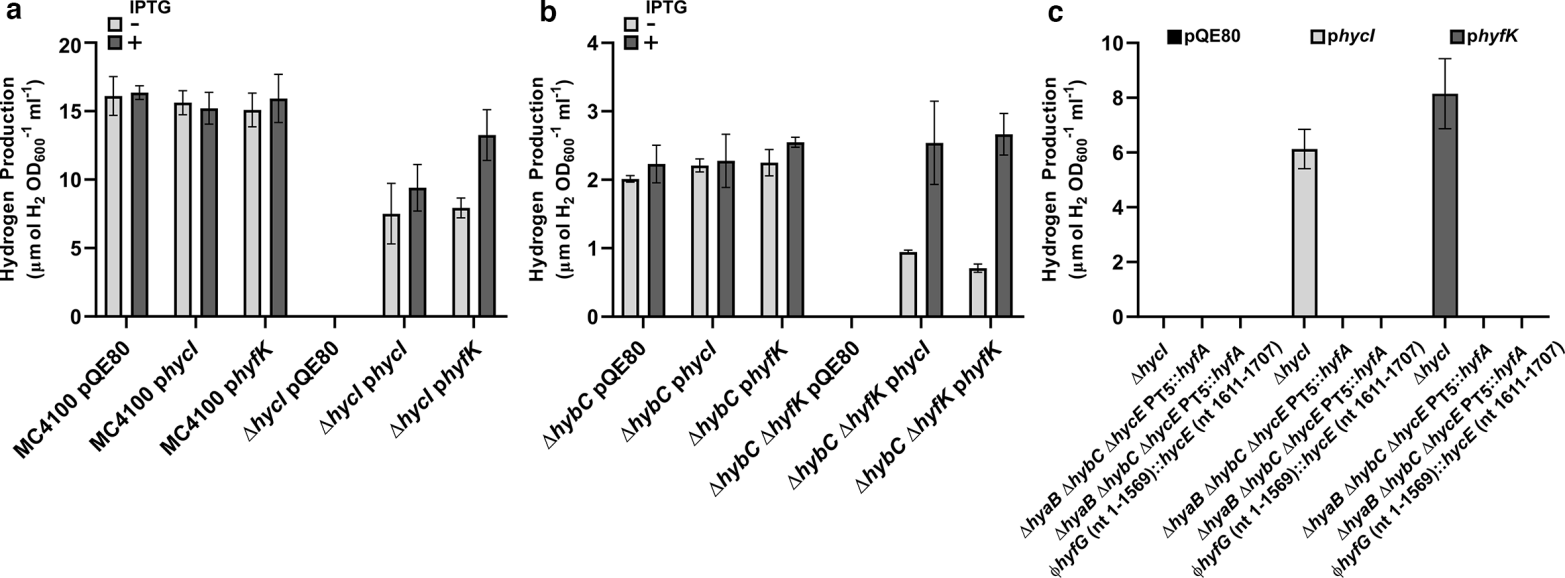
*
P. atrosepticum* HyfK can activate *E. coli* Hyd-3, and *E. coli* HycI can activate *P. atrosepticum* Hyd-4. (a) *E. coli* strains MC4100 (FHL-1^+^) and MGE1dI (Δ*hycI*) were transformed separately with plasmids harbouring *hycI^Ec^*, *hyfK^Pba^* or a vector control (pQE80). Strains were grown anaerobically in LB medium supplemented with 0.2 % (w/v) formate, ampicillin and 1 mM IPTG where indicated (+) for 24 h at 37 °C. (b) *P. atrosepticum* strains PH002 (Δ*hybC*, FHL-2^+^) and PH006 (Δ*hybC*, Δ*hyfK*) were transformed with plasmids containing *hycI^Ec^*, *hyfK^Pba^* or pQE80. Strains were grown anaerobically in low-salt LB (LSLB) medium supplemented with ampicillin and 1 mM IPTG where indicated (+) for 48 h at 24 °C. (c) *E. coli* strains MGE1dI (Δ*hycI*), FTE003 (Δ*hyaB*, Δ*hybC*, Δ*hycE*, P_T5_::*hyfA*) and FTE004 (Δ*hyaB*, Δ*hybC*, Δ*hycE*, P_T5_::*hyfA*, ϕ*hyfG::hycE*) were transformed with plasmids containing *hycI^Ec^*, *hyfK^Pba^* or a vector control (pQE80). Strains were grown anaerobically in LB medium supplemented with 0.8 % (w/v) glucose, ampicillin and 1 mM IPTG for 16 h at 37 °C. In all cases, H_2_ headspace samples were extracted and analysed by gas chromatography (Shimadzu GC2014 using a 5A molecular packed column with thermal conductivity detection). Data was normalized by OD_600_ and culture volume. Error bars represent sd (*n*=3).

*P. atrosepticum* SCRI1043 is a γ-Proteobacterium that contains an active FHL-2 and Hyd-4 encoded by a *hyf* operon (Fig. 1a), but no FHL-1 or Hyd-3 isoenzyme [28]. Unlike the *E. coli hyf* operon, the *P. atrosepticum* SCRI1043 *hyf* operon encodes HyfK (HyfK*_Pa_*, Fig. 1a), which shares 74 % overall sequence identity with *E. coli* HycI (HycI*_Ec_*). Due to this sequence similarity it was considered that these endopeptidases could be tested for their ability to activate either the Hyd-3-type and the Hyd-4-type hydrogenase. To begin, both *hycI_Ec_* and *hyfK_Pa_* genes were cloned separately in to pQE80L (Amp^R^) expression vectors using standard PCR and molecular cloning techniques. Both plasmids, and a vector control, were used to transform the *E. coli* Δ*hycI* strain MGE1dI (Table 1). *E. coli* MGE1dI is based on MG059e1 (as MG1655, *hycE* ^His^ [25]) but carries an unmarked in-frame deletion in *hycI*. The transformed *E. coli* strains were grown in triplicate 5 ml LB 0.2 % (w/v) formate cultures, with or without addition of 1 mM IPTG, anaerobically in sealed Hungate tubes, for 24 h at 37 °C, before GC analysis of the culture headspace. The MGE1dI (Δ*hycI*) strain of *E. coli *, containing empty vector control, displayed no physiological FHL-1 activity and did not evolve H_2_ gas under fermentative conditions (Fig. 2a). However, incorporation of either *hycI_Ec_* or *hyfK_Pa_* in the *E. coli* Δ*hycI* strain rescued H_2_ production (Fig. 2a). These data demonstrate that the *hyfK_Pa_* gene product can facilitate the maturation of the *E. coli* Hyd-3 enzyme.

To study the role of maturation proteases in the activation of Hyd-4/FHL-2, *P. atrosepticum* SCRI1043, which contains active FHL-2, was studied [28]. First, a genetic approach was taken to assess the role of *hyfK* in hydrogen production. A *P. atrosepticum* double-mutant strain was constructed, using an allele exchange protocol [28], which carried both Δ*hybC* and Δ*hyfK* in-frame deletions (PH006, Table 2 and Supplementary Material). The Δ*hybC* deletion removes all Hyd-2 activity leaving Hyd-4 as the only active hydrogenase in the bacterium [28]. Next, the *P. atrosepticum* Δ*hybC* Δ*hyfK* double-mutant (PH006), together with the *P. atrosepticum* PH002 parent strain (Δ*hybC* only), were separately transformed with the pQE80 plasmids containing either *hycI_Ec_* or *hyfK_Pa_*, or the empty vector as a control. The transformed *P. atrosepticum* strains were then grown in triplicate 5 ml low salt LB cultures (5 g l^−1^ NaCl as opposed to the commonly used 10 g l^−1^), with or without addition of 1 mM IPTG, in sealed Hungate tubes, fermentatively for 48 h at 24 °C, before GC analysis of the headspace gases. The *P. atrosepticum* PH002 parent strain (Δ*hybC*) was able to generate H_2_ gas under all conditions (Fig. 2b). However, the Δ*hybC* Δ*hyfK* double-mutant was incapable of producing any H_2_ gas in this experiment when carrying an empty vector (Fig. 2b). This shows the *hyfK_Pa_* protease gene is essential for FHL-2 and Hyd-4 activity in *P. atrosepticum* SCRI1043. Moreover, the *P. atrosepticum* Δ*hybC* Δ*hyfK* double-mutant strain was clearly rescued for H_2_ production by inclusion of either *hycI_Ec_* or *hyfK_Pa_* (Fig. 2b). These data demonstrate that Hyd-4 isoenzymes do require a maturation step for successful biosynthesis and they also suggest that, in the rare cases where an organism has the capability to produce both FHL-1 and FHL-2, that a single copy of *hycI* should be sufficient for this task.

**Table 2.** Mutagenesis of the *P. atrosepticum hyf* operon

*P. atrsosepticum* strain	Relevant genotype	Source	H_2_ production
**SCRI1043**	wild-type	[48]	Positive
**PH002**	as SCRI1043, Δ*hybC*	[28]	Positive
**PH006**	as SCRI1043, Δ*hybC*, Δ*hyfK*	This Work	Negative

*P. atrosepticum strains were grown under anaerobic fermentative conditions in low salt (LS) LB medium supplemented with 0.8 % (w/v) d-glucose at 24 °C for 48 h. Production of molecular H_2_ in the culture headspace was determined by GC [28]

GC, Gas Chromatography; LB, Lysogeny Broth; LSLB, Low Salt Lysogeny Broth.

This compatibility of HycI*_Ec_* and HyfK*_Pa_* for activation of either *E. coli* Hyd-3 or *P. atrosepticum* Hyd-4 points strongly towards the idea that *E. coli* HycI should be capable of maturation of the endogenous Hyd-4 found in *E. coli *. In one final attempt to observe Hyd-4 activity in *E. coli *, the *hycI_Ec_* and *hyfK_Pa_* encoding plasmids, and a vector control, were each used to transform the *E. coli* FTE003 and FTE004 strains encoding the HyfG::HycE fusion proteins (Table 1). All strains were grown in triplicate 5 ml LB 0.8 % (w/v) glucose cultures, with addition of 1 mM IPTG, in sealed Hungate tubes, for 16 h at 37 °C, before GC analysis. Hydrogen production was only detected in the control strains (Fig. 2c), demonstrating that cellular levels of a maturation protease is not the sole factor limiting Hyd-4 activity in *E. coli *.

This work presents the first demonstration of cross-species complementation by hydrogenase maturation endopeptidases, highlighting the close evolutionary relationship between group 4 [NiFe]-hydrogenases and demonstrating the critical importance of the HycI-type protease in the biosynthesis of these enzymes. These data are in line with studies showing a endopeptidase for a group 1D hydrogenase was able to activate a different group 1D enzyme within the same organism (* Salmonella enterica *) [34], and one endopeptidase was able to activate both a group 3B hydrogenase and group 4D hydrogenase within the same organism (* Thermococcus kodakarensis *) [35].

Though the proteolytic maturation schedule for [NiFe]-hydrogenases is now dogma, there are known and emerging variations on the canonical pathway for large subunit biosynthesis. Protelolytic processing is not required for all [NiFe]-hydrogenases, such as examples of the H_2_-sensing, Ech- and CODH-linked hydrogenases [36–39]. Indeed, recent genetic engineering work showed that removal of the C-terminal assembly peptide from the membrane bound hydrogenase (MBH) in *Cupriavidus necator* (* Ralstonia eutropha *) did not disrupt cofactor insertion and resulted in no loss of hydrogenase-specific activity [40]. Given that in *S. enterica* a maturation protease was found to retain the ability to recognize and bind to a large subunit completely lacking the maturation peptide [34], perhaps it should be considered that the maturation protease has a role in hydrogenase biosynthesis beyond the simple cleavage of the C-terminal extension. This could certainly be tested in the *C. necator* system [40] by deleting the gene encoding the processing protease (HoxM [41]) in the stain already lacking the hydrogenase assembly peptide and observing any changes to hydrogenase activity.

It is becoming increasingly clear that the C-terminal assembly peptide may not be the key recognition motif for the protease [34, 42]. Early work showed that swapping of the *E. coli* HycE (Hyd-3) assembly peptide for that of HybC (Hyd-2) led to a ‘dead-end’ fusion protein that could not be processed by any maturation protease tested [43]. While more recently, swapping the HybC (Hyd-2) assembly peptide for that of HyaB (Hyd-1) did not lead to any changes in the requirement for the Hyd-2-specific protease (HybC) for maturation [42, 44].

In conclusion, this work has demonstrated that group 4 [NiFe]-hydrogenases require a functional *hycI*-like accessory gene for correct biosynthesis. A model bacterium (* P. atrosepticum* SCRI1043), which contains an active Hyd-4 and FHL-2 as the only formate hydrogenlyase activity, required the presence of the native *hyfK* gene product for maturation. The *E. coli hycI* gene could substitute for *P. atrosepticum hyfK* if supplied on a plasmid, providing an explanation of why it is that in rare examples of organisms that contain both an FHL-1 and an FHL-2 only one copy of a *hycI*-like gene is conserved.

## Supplementary Data

Supplementary material 1
