# Supplementary material for: Activation of a [NiFe]-hydrogenase-4 isoenzyme by maturation proteases
Source: Microbiology (Reading). 2020 Jul 30;166(9):854–60. doi: 10.1099/mic.0.000963 (PMC7654741; doi:10.1099/mic.0.000963)
Supplement: Supplementary material 1 [file mic-166-854-s001.pdf]

Activation of a [NiFe]-hydrogenase-4 isoenzyme by maturation proteases.

Alexander J. Finney, Grant Buchanan, Tracy Palmer, Sarah J. Coulthurst and Frank Sargent

SUPPLEMENTARY INFORMATION

Construction of *E. coli* strains

All strains are listed in Table 1 of the main manuscript. This research was based on *E. coli* K-12 using both MG1655 [1] and MC4100 [2] as the parental strains. The MG056G1 strain (as MG1655, *hyfG*^{His}) was constructed using primers HyfGupXba (5'-GCGCTCTAGACCTGGCGGGCGCTTCCACTATAGTCC-3') and HyfGupBam (5'-GCGCGGATCCGTGATGGTGATGGTGATGGTGATGGTGATGCCCTTCCATTGAAAGG GCATAATAAACC-3') to amplify a sequence 600 bp upstream of the *hyfG* Gly-85 codon including a 10-His sequence, and primers HyfGdownBam2 (5'-GCGCGGATCCGCCGAAAAATGCTGGATTGTGGTGAA GGCGCTGG-3') and HyfGdownKpn2 (5'-GCGCGGTACCCCGGTATCAAAACCAACGAAATGGCAGGAG AGGCC-3') to amplify a 600 bp fragment downstream and in-frame with the *hyfG* Ala-86 codon. Once assembled together on pMAK705 in a three-way XbaI/BamHI/KpnI digestion and ligation [3], the resultant *hyfG*^{His} allele was verified by sequencing and moved on to the chromosome of MG1655 by homologous recombination using the method of Hamilton *et al*. [3]. Successful chromosomal integration of the 10-His allele was done by selection for chloramphenicol-sensitive colonies, before checking by PCR and successful digestion of the *hyfG* product with BamHI. DNA sequencing confirmed all mutant sequences.

For promoter engineering, the P_{hyc}::*hyfA* and P_{T5}::*hyfA* alleles were specifically designed to incorporate the *E. coli hyc* promoter and the phage T5 transcriptional promoter upstream of the *hyfA* gene on the bacterial chromosome. The alleles were assembled on to pMAK705 [3] using Gibson assembly techniques ready to be transferred to the chromosome of any chosen *recA*$^+$ host strain. The P_{hyc}::*hyfA* allele was incorporated in to MG056G1 to give strains AF01. This new strain (AF01) was then extensively modified, first with the genetic removal of Hydrogenase-3 activity using deletion alleles and methodology recently described [4], yielding strains AF02 and AF03. These mutants were then further modified by the inclusion of a Δ*hyaB* allele designed to remove the Hydrogenase-1 catalytic subunit [5], finally resulting in the MG1655 derivatives AF04 and AF06.

The *E. coli* FTD147 strain (based on MC4100 but deleted for the genes encoding the catalytic subunits of Hydrogenases- 1, -2 and -3 [6]) was modified by pMAK705-based homologous replacement of the native *hyf* promoter with that of the *E. coli hyc* operon (new strain AF05) or the stronger phage T5 promoter (resulting in strain FTE003). The FTE003 strain was further engineered by transferring in the *hyfG*^{His} allele encoding 10-His tagged Hyd-4, resulting in FTE005.

Finally, an allele that would genetically fuse the mature sequence of *E. coli* HyfG to the C-terminal assembly peptide of *E. coli* HycE was designed. A synthetic biology Gibson assembly approach was taken to construct the fusion allele on pMAK705. Three fragments of DNA were amplified by PCR. First, a 600 bp fragment of *hyfG* terminating precisely at nucleotide 1569; second a small fragment of DNA encoding *hycE* nucleotides 1611-1707; and then a 600 bp fragment downstream of *hyfG*. This

allowed assembly of a ϕ*hyfG*::*hycE* that was used to modify five strains (AF03; AF05; FTE003; FTE005 and FTD147) resulting in the new mutants FTE001; FTE002; FTE004; FTE006; and FTE007.

This work also features an *E. coli* K-12 Δ*hycI* strain. This belongs to a series of mutants described by McDowall *et al*. [7]. The MG1655 parent strain was modified to contain a chromosomal *hycE*^{His} allele and named MG059e1 [8]. This mutant was then further modified by the construction of an unmarked, in-frame Δ*hycI* allele on pMAK705, which was transferred to the chromosome by homologous recombination [3]. The new *hycE*^{His} Δ*hycI* strain was named MGE1dI as it is derived from the MG1655 parent strain, contains the His-tag allele from MG059e1, and is deleted for *hycI*.

Construction of *P. atrosepticum* strains

All strains are shown in Table 2 of the main manuscript. The parental strain used in this study was *P. atrosepticum* SCRI1043 [9] and the previously-constructed PH002 derivative lacking the *hybC* gene encoding the catalytic subunit of Hyd-2 [10]. The in-frame deletion mutant of *hyfK* was constructed using pKNG101 suicide vector in *E. coli* strain CC118λpir [11, 12]. Oligonucleotide primers to upstream and downstream regions (≥600 bp) of the *hyfK* gene were carefully designed to preserve natural ribosome binding sites, initiation and termination codons, and so generate an unmarked, in-frame deletion allele. The fragments were amplified by PCR and cloned in to the pKNG101 vector using Gibson assembly (HiFi Assembly, New England Biolabs). The *E. coli* CC118λpir strain was transformed with the pKNG101-Δ*hyfK* plasmid before it, an *E. coli* HH26 pNJ5000 helper strain, and the PH002 Δ*hybC P. atrosepticum* strain were grown separately in rich medium, with antibiotics as necessary. Equal volumes of the stationary phase cultures were then mixed together and 30 µL was spotted on a non-selective rich medium agar plate for 24 hours at 24˚C. Spots were then suspended in 1 ml rich medium before *P. atrosepticum* harbouring the pKNG101-Δ*hyfK* plasmid were initially selected for by plating on minimal medium agar with streptomycin (100 µg/ml). Single colonies were then struck on minimal media agar plates with streptomycin for a further 14 hours at 24 °C. Co-integrants were then grown to stationary phase in rich medium with no selection before the culture was diluted 1/500 with phosphate buffer and 30 µL plated on minimal medium agar with sucrose. Surviving colonies were the patch screened to ensure sensitivity to streptomycin before colony PCR was used to confirm deletion of the *hyfK* gene.

Supplementary References

1. **Blattner FR, Plunkett G, 3rd, Bloch CA, Perna NT, Burland V et al.** The complete genome sequence of *Escherichia coli* K-12. *Science* 1997;277(5331):1453-1462.

2. **Casadaban MJ, Cohen SN**. Lactose genes fused to exogenous promoters in one step using a Mu-lac bacteriophage: *in vivo* probe for transcriptional control sequences. *Proceedings of the National Academy of Sciences of the United States of America* 1979;76(9):4530-4533.

3. **Hamilton CM, Aldea M, Washburn BK, Babitzke P, Kushner SR**. New method for generating deletions and gene replacements in *Escherichia coli*. *Journal of Bacteriology* 1989;171(9):4617-4622.
4. **Pinske C, Sargent F**. Exploring the directionality of *Escherichia coli* formate hydrogenlyase: a membrane-bound enzyme capable of fixing carbon dioxide to organic acid. *Microbiology Open* 2016;5(5):721-737.
5. **Dubini A, Pye RL, Jack RL, Palmer T, Sargent F**. How bacteria get energy from hydrogen: a genetic analysis of periplasmic hydrogen oxidation in *Escherichia coli*. *International Journal of Hydrogen Energy* 2002;27(11-12):1413-1420.
6. **Redwood MD, Mikheenko IP, Sargent F, Macaskie LE**. Dissecting the roles of *Escherichia coli* hydrogenases in biohydrogen production. *FEMS Microbiology Letters* 2008;278(1):48-55.
7. **McDowall JS, Hjersing MC, Palmer T, Sargent F**. Dissection and engineering of the *Escherichia coli* formate hydrogenlyase complex. *FEBS Letters* 2015;589:3141-3147.
8. **McDowall JS, Murphy BJ, Haumann M, Palmer T, Armstrong FA et al.** Bacterial formate hydrogenlyase complex. *Proceedings of the National Academy of Sciences of the United States of America* 2014;111(38):E3948-3956.
9. **Bell KS, Sebaihia M, Pritchard L, Holden MT, Hyman LJ et al.** Genome sequence of the enterobacterial phytopathogen *Erwinia carotovora* subsp. atroseptica and characterization of virulence factors. *Proceedings of the National Academy of Sciences of the United States of America* 2004;101(30):11105-11110.
10. **Finney AJ, Lowden R, Fleszar M, Albareda M, Coulthurst SJ et al.** The plant pathogen *Pectobacterium atrosepticum* contains a functional formate hydrogenlyase-2 complex. *Molecular Microbiology* 2019;112(5):1440-1452.
11. **Kaniga K, Delor I, Cornelis GR**. A wide-host-range suicide vector for improving reverse genetics in Gram negative bacteria: inactivation of the *blaA* gene of *Yersinia enterocolitica*. *Gene* 1991;109(1):137-141.
12. **Coulthurst SJ, Lilley KS, Salmond GP**. Genetic and proteomic analysis of the role of *luxS* in the enteric phytopathogen, *Erwinia carotovora*. *Molecular Plant Pathology* 2006;7(1):31-45.
